# A Systematic Comparative Study of Potato, Sweet Potato, and Tapioca Starches: From Molecular Architecture to Processing Performance

**DOI:** 10.3390/gels12100939

**Published:** 2026-10-20

**Authors:** Guohua Fan, Songnan Li, Xiang Gao, Hong Li, Jiaxin Li, Hui Wang, Jianghui Li, Yuandong Wang, Huiyong Shen, Xiaomin Feng

**Affiliations:** 1 Institute of Sorghum Research, Shanxi Agricultural University, Jinzhong 030600, China; 2 Joint International Research Laboratory of Agriculture and Agri-Product Safety of the Ministry of Education of China, Institutes of Agricultural Science and Technology Development, Yangzhou University, Yangzhou 225009, China; 3 College of Agriculture, Shanxi Agricultural University, Jinzhong 030801, China; 4 Institute of Cotton Research, Shanxi Agricultural University, Yuncheng 044000, China; 5 Agricultural Water Development Center of Hequ, Xinzhou 034000, China

**Keywords:** chain-length, gelatinization behavior, pasting viscosity, gel texture, digestibility

## Abstract

Potato, sweet potato, and tapioca starches are three of the most commercially important root and tuber starches, yet they exhibit markedly different molecular structures, which directly determine their distinct processing performances such as gelatinization behavior, pasting viscosity, gel texture, and digestibility. Therefore, this study systematically compared the molecular architecture and processing performances of potato starch (PS), sweet potato starch (SPS), and tapioca starch (TS). Chain-length profiling revealed that, compared with PS, both SPS and TS were enriched in short amylopectin chains but depleted in medium- and long-chain fractions. In terms of pasting behaviors, PS gave higher peak, trough, and final viscosities but a lower setback value than SPS and TS. Regarding thermal properties, PS exhibited a higher enthalpy change (13.53 J/g) than SPS and TS, whereas TS showed the highest onset temperature (66.10 °C). For gel texture, PS had the greatest hardness (519.31 g), gumminess (477.98 g), and chewiness (465.49 g). Importantly, regarding digestibility, SPS exhibited the highest level of resistant starch (65.66%) and the lowest level of rapidly digestible starch (10.86%), whereas PS showed the lowest resistant starch (56.36%) and the highest rapidly digestible starch (20.36%). Collectively, these findings are critical for the rational selection of starches for specific food and industrial uses to broaden their application potential.

## 1. Introduction

Root and tuber crops are defined as agricultural plants that accumulate edible biomass and nutrients in underground storage organs, including roots, corms, and tubers [1]. As staple food sources, these crops occupy a position of critical importance within global agricultural systems, particularly in developing regions, where they sustain hundreds of millions of people. They contribute substantially to food security, nutritional provision, and household income, especially for low-income farmers and food-insecure communities. A key nutritional advantage of root and tuber crops lies in their ability to serve as cost-effective sources of dietary energy, owing to their high carbohydrate content [2]. Among these, potato (*Solanum tuberosum* L.), sweet potato (*Ipomoea batatas* (L.) Lam.), and cassava (*Manihot esculenta* Crantz), the latter being the botanical source of tapioca starch, are among the most widely cultivated and economically important starch crops worldwide [3]. In recent years, these three starches collectively accounted for ~36% of the starch used in gel and hydrocolloid applications, with potato starch, sweet potato starch, and tapioca starch contributing ~12%, ~6%, and ~18%, respectively [4]. Potato ranks as the third most important food crop worldwide in terms of human consumption, following only rice and wheat [5]. The Food and Agriculture Organization has consistently recommended potato as a crop of strategic importance for food security, particularly as the world confronts the dual challenges of population growth and food supply disruptions. Sweet potato is of high economic value owing to its versatile applications and multiple beneficial properties. It serves as a reliable source of income for smallholder farmers, due to its high productivity, low input requirements, and short growth cycle [6]. Furthermore, growing industrial demand for sweet potato in food processing, including flour, snacks, and other processed products, has further bolstered its economic importance. Tapioca stands as one of the most strategically significant staple crops in the tropical and subtropical regions of the world. Its remarkable adaptability to marginal soils and drought-prone environments, coupled with its high carbohydrate yield per unit area, underpins its critical role in food security strategies across developing regions [7].

In root and tuber crops, starch constitutes the major fraction of dry matter (typically 50–80% in sweet potato, and similar high proportions in potato and tapioca) [8]. Its multi-scale structure (from granule morphology to molecular chain architecture) directly dictates processing performance, nutritional value, and industrial applicability. Understanding starch is therefore equivalent to understanding the crop’s core value as food and raw material. Among tuber starches from potato, sweet potato, and tapioca, there are significant differences in crystalline type (potato: B-type; sweet potato and tapioca: A-type), gelatinization behavior, pasting viscosity, freeze–thaw stability, and digestibility [9]. These differences determine their specific uses; thus, elucidating these structure–function relationships are essential for precise and value-added utilization.

Potato starch (PS) is widely used as a functional ingredient in bakery and confectionery products, and plays critical roles in industrial applications such as paper manufacturing and pharmaceuticals [10]. Moreover, it has gained increasing attention as a promising material for active food packaging films and nanocomposites [11], underscoring its broadening versatility. Sweet potato starch (SPS) is characterized by a high resistant starch content and a low glycemic index relative to other starch-based foods [12]. SPS with thicker semicrystalline lamellae showed a relatively stronger retrogradation tendency, and can be used to develop starch-based foods with desirable gel structure [13]. Tapioca starch (TS) exhibits distinct advantages, including low production cost, high purity, low amylose content, and high paste viscosity [14]. It is extensively utilized in the food industry as a key ingredient in sweeteners (e.g., high-fructose syrup), tapioca pearls, bakery and pastry goods [7]. Moreover, potato, sweet potato, and tapioca starches have been extensively utilized in gel-based food applications. PS gels are valued for their high viscosity and transparency, and have been applied in hydrogel foods and 3D food printing. SPS gels are widely used in gluten-free starch noodles, vermicelli, confectionery, and jellies. TS gels, owing to their soft and transparent appearance, are commonly used in desserts such as cendol and starch pearls in bubble tea, as well as in texture-modified foods for dysphagia management.

Although the structural characteristics and processing properties of the three tuber starches (PS, SPS, and TS) have been individually investigated, a systematic study that establishes explicit correlations between their molecular structural features and processing performance has not yet been reported. In this study, PS, SPS, and TS were obtained from a commercial supplier with corresponding national variety standards, respectively. Their total starch content and molecular structure, specifically the chain-length distributions (CLDs) of amylose and amylopectin, were first determined. Subsequently, a comprehensive characterization of granular properties and functional properties (thermal behavior, pasting viscosity, textural properties, freeze–thaw stability, and in vitro digestibility) was conducted. The findings of this study are expected to provide a theoretical basis for the further processing and utilization of tuber starches.

## 2. Results and Discussion

### 2.1. Total Starch Content and Chroma Values

Table 1 shows the total starch content and chroma values of three tuber starches (PS, SPS, and TS). The total starch contents of PS, SPS, and TS, determined on a dry-weight basis, were 94.03%, 92.14%, and 92.56%, respectively. Although PS exhibited a slightly higher value, no statistically significant difference was observed among the three starches, which is consistent with previous reports [15,16]. Among the samples, TS displayed the highest lightness value (L* = 98.30), which differed significantly from those of PS and SPS. Conversely, SPS gave the greatest chromaticity coordinates a* and b*, and these values were also significantly different from those of PS and TS. This phenomenon indicates that SPS may contain residual pigments, which are related to its variety source and extraction process [17].

### 2.2. Chain-Length Distributions (CLDs) of Amylose and Amylopectin

**Figure 1:**
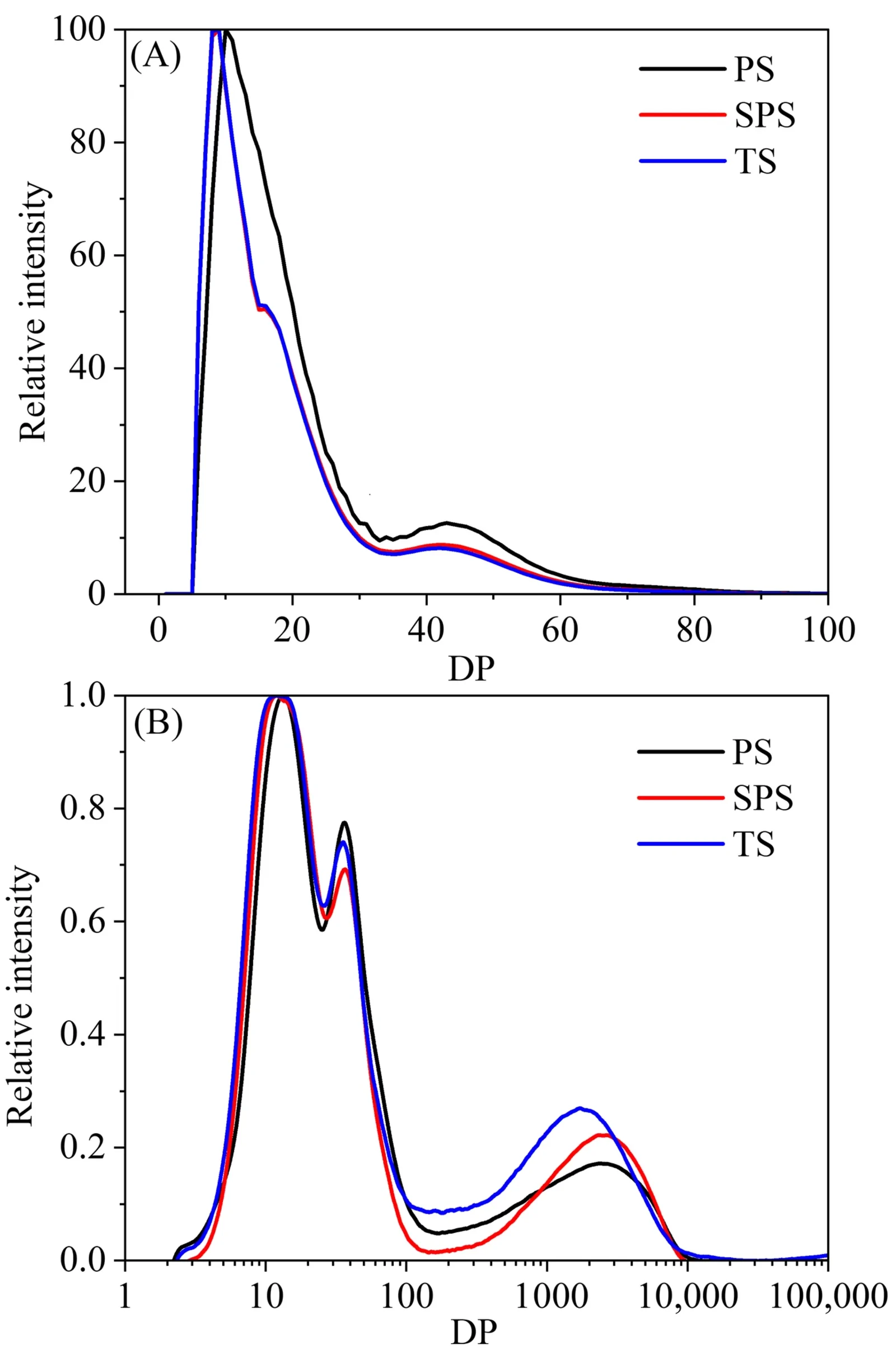
Fluorophore-assisted carbohydrate electrophoresis (**A**) and size-exclusion chromatography (**B**) curves from degree of polymerization (DP) classification of three tuber starches (PS, SPS, and TS).

CLDs of amylopectin and amylose in three tuber starches (PS, SPS, and TS) were analyzed by fluorophore-assisted carbohydrate electrophoresis (FACE) and size exclusion chromatography (SEC), respectively. Figure 1A shows the FACE curves of three tuber starches (PS, SPS, and TS). Compared to SPS and TS, PS exhibits a completely different CLD trend of amylopectin. Figure 1B shows the SEC curves of three tuber starches (PS, SPS, and TS). The CLD of amylose in the PS, SPS, and TS shows significant differences. The above differences in molecular chain-length (CL) structure were further resolved in detail using defined degree of polymerization (DP) ranges. The CLDs of amylopectin and amylose were classified into several fractions according to the literature [18], with DP intervals as follows: 6 ≤ DP ≤ 12, 13 ≤ DP ≤ 24, 25 ≤ DP ≤ 36, 37 ≤ DP < 100, 100 ≤ DP < 500, 500 ≤ DP < 5000, and 5000 ≤ DP ≤ 20,000. The relative proportion of each fraction was calculated by dividing the integrated area under the curve for the corresponding DP range by the total area under the curve, using either FACE or SEC profiles, according to established methods [15,16].

The CLDs of amylopectin and amylose for the three tuber starches are summarized in Table 2. For amylopectin, PS exhibited notably higher proportions of medium (DP 13–24, 40.80%), medium-long (DP 25–36, 10.78%), and long (DP 37–100, 15.84%) chains compared with SPS and TS. In contrast, SPS and TS showed very similar CLD profiles, both being enriched in short chains (DP 6–12) and depleted in longer fractions. This marked difference suggests that PS possesses a greater abundance of longer external chains, which may influence its pasting and retrogradation behavior. Longer amylopectin chains are known to promote intermolecular associations and crystalline lamellae stability, potentially leading to higher gelatinization temperatures and a greater tendency for retrogradation upon cooling [19]. Conversely, the predominance of short chains in SPS and TS may contribute to weaker gel networks and improved freeze–thaw stability, as shorter branches impede the formation of stable crystalline junctions [20].

For amylose, the fractions were divided into short (DP 100–500), medium (DP 500–5000), and long (DP 5000–20,000) categories. The total amylose content ranged from 21.98% (PS) to 28.11% (TS), indicating a marked variety difference. Notably, SPS displayed the highest proportion of medium-length amylose chains (DP 500–5000) among the three starches. Medium-length amylose chains are generally considered to be more effective in forming double-helical structures and enhancing gel strength, as they possess an optimal balance between chain mobility and entanglements [21]. The elevated medium-chain amylose fraction in SPS may therefore account for its superior gel-forming capacity and textural properties, which are desirable for noodle and snack applications. In contrast, the lower amylose content and a relatively higher short-chain amylose fraction in PS may lead to weaker gels but could also reduce retrogradation, thus extending shelf-life in processed foods. The distinct CLD patterns observed among the three starches highlight the critical role of molecular structure in determining functional performance, and provide a structural basis for tailoring starch modifications or selecting appropriate cultivars for specific food and industrial applications.

### 2.3. Pasting RVA Parameters

**Figure 2:**
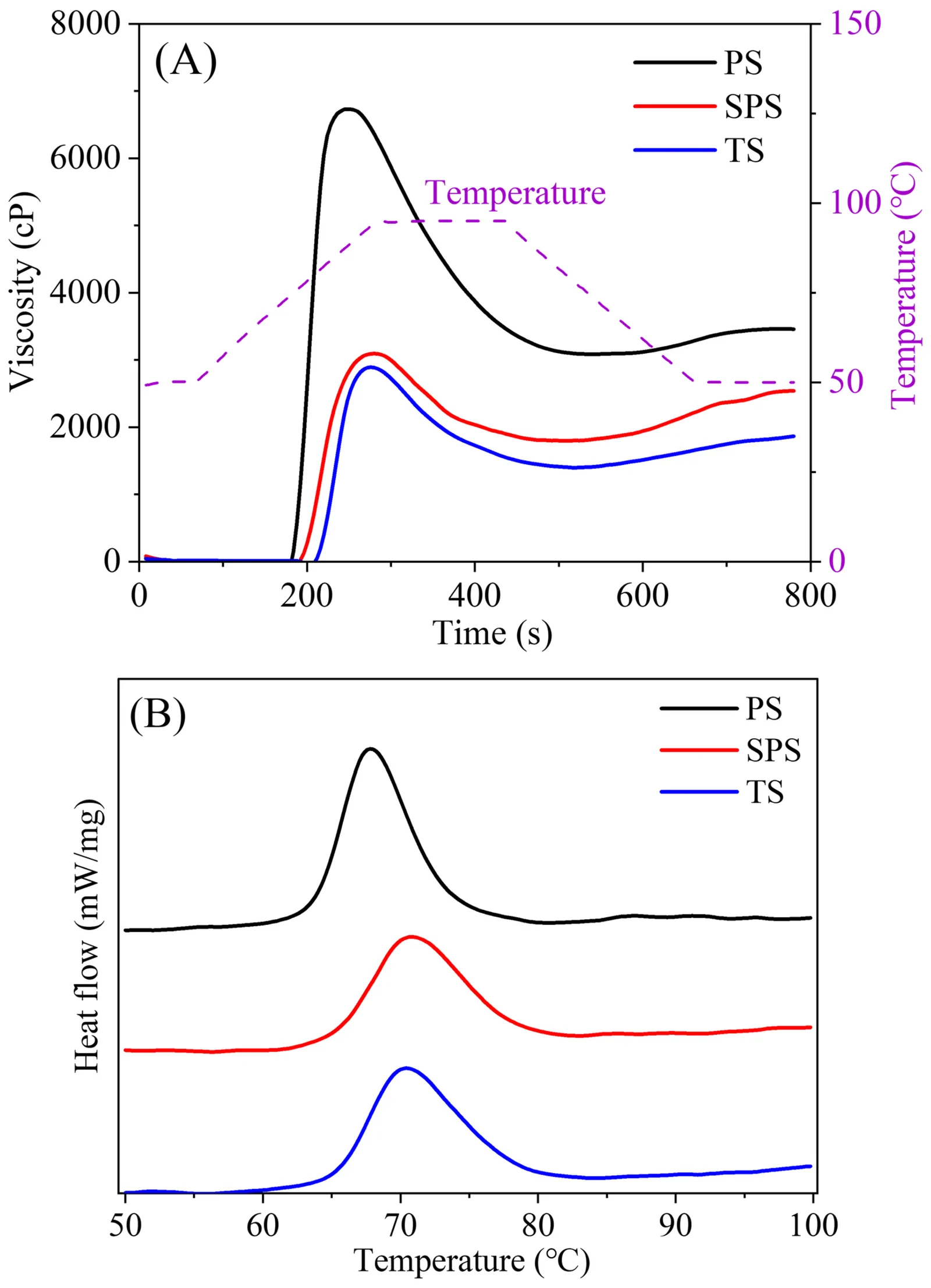
Rapid viscosity analyzer (RVA; (**A**)) and differential scanning calorimetry (DSC; (**B**)) curves of three tuber starches (PS, SPS, and TS).

Figure 2A exhibits the rapid viscosity analyzer (RVA) curves of three tuber starches (PS, SPS, and TS). Consistent with previous studies [15,22], notable variations were observed in the pasting profiles among the three tuber starches (Table 3). PS exhibited the highest peak viscosity (PV, 6876 cP), while TS showed the lowest (2871 cP), with sweet potato starch (SPS) giving an intermediate value (3134 cP). This trend is likely attributable to the substantially higher proportion of amylopectin medium chains (DP 13–24) in PS compared with SPS and TS (Table 3), as a positive correlation between the abundance of medium-length amylopectin chains and PV has been well documented [23]. These chains facilitate granule swelling by promoting intra-granular water absorption and maintaining structural integrity during heating, thereby enhancing the initial viscosity. Conversely, the lower PV values of SPS and TS may be associated with their higher contents of short amylopectin chains (DP 6–12), which tend to form less stable crystalline lamellae and restrict granule expansion. It should be noted that the high pasting viscosity of PS cannot be attributed solely to its chain-length distribution. Other source-specific factors, such as phosphate monoester content (not measured in this study), granule size (Figure S1), and minerals-related ash content (Table S1), may also contribute substantially to granule swelling and paste viscosity [24]. The observed differences in PV among starches may reflect a combination of these and other factors. Further studies are required to quantify the specific contributions of these structural and molecular factors.

The trough viscosity (TV) and final viscosity (FV) followed a similar order: PS > SPS > TS. The notably higher TV and FV of PS indicate a thicker paste matrix after heating and cooling, which is consistent with its high amylopectin chain length and low amylose content. The breakdown viscosity (BD = PV − TV), which reflects the starch paste stability under combined heating and mechanical shear, was highest for PS (3701 cP), followed by TS (1495 cP) and SPS (1320 cP). The extremely high BD of PS suggests that its paste is less stable under combined heating and mechanical shear, probably because the abundant medium-chain amylopectin and low amylose lead to a fragile granule structure that collapses rapidly upon agitation [25].

The setback viscosity (SB = FV − TV), an empirical short-term reassociation indicator, followed a different order: SPS (713 cP) > TS (504 cP) > PS (330 cP). The substantially higher SB of SPS suggests that its paste has the strongest propensity to reassociate upon cooling, forming a firmer gel. This behavior is consistent with our CLD result, which showed that SPS possesses the highest proportion of medium-length amylose chains (DP 500–5000). Such chains are known to be more effective in forming double-helical structures and intermolecular junctions during retrogradation, thereby enhancing gel strength [15].

Overall, the RVA parameters clearly demonstrate that the pasting behaviors of the three tuber starches are governed not only by total amylose content but also, and more importantly, by the fine chain-length distributions of both amylopectin and amylose. The distinct profiles of PS (high PV and BD, low SB), SPS (moderate PV and BD, high SB), and TS (low PV and BD, moderate SB) provide valuable guidance for selecting appropriate starches for specific food applications: PS for thickeners requiring high viscosity, SPS for gelling agents with strong retrogradation, and TS for products needing low viscosity and neutral taste.

### 2.4. Gelatinization DSC Parameters

Figure 2B presents the differential scanning calorimetry (DSC) curves of three tuber starches (PS, SPS, and TS), and their DSC characteristic values are shown in Table 4. TS showed the highest onset temperature (*T*_o_ = 66.10 °C), whereas SPS gave the lowest (62.97 °C) and PS was intermediate (64.27 °C); however, the differences in *T*_o_ were not statistically significant due to the relatively large standard deviation for SPS. For both peak temperature (*T*_p_) and conclusion temperature (*T*_c_), SPS and TS displayed significantly higher values (*T*_p_ = 70.93 °C and 70.53 °C; *T*_c_ = 77.23 °C and 77.13 °C, respectively) compared with PS (*T*_p_ = 67.97 °C, *T*_c_ = 73.00 °C). This may be partly attributed to the higher amylose content of TS (28.11%) relative to PS and SPS, as amylose molecules can form complexes with lipids or interact with amylopectin to stabilize crystallites [26]. Additionally, the chain-length distributions of amylopectin may play a crucial role: PS is characterized by a higher proportion of medium and long amylopectin chains (DP 13–24, 25–36 and 37–100), which generally promote the formation of thicker crystalline lamellae with higher melting temperatures [27]. Conversely, PS exhibited the highest enthalpy change (Δ*H*; 13.53 J/g), followed by TS (11.24 J/g) and SPS (10.69 J/g). The greater Δ*H* for PS indicates suggests a larger energy input required to disrupt ordered double-helical structures under the selected hydration conditions [28], despite its lower gelatinization temperatures.

Altogether, the DSC data indicate that PS exhibits a greater Δ*H*, suggesting a larger amount of ordered double-helical structures, but lower thermal stability (lower *T*_p_ and *T*_c_) than SPS and TS. These differences cannot be attributed to amylose content alone, because PS and SPS have essentially identical amylose contents (21.98% and 22.05%, respectively). Instead, they likely reflect a combination of crystalline type (Figure S2), amylopectin chain-length distribution, and, in the case of TS, its higher amylose content (28.11%). The findings provide a structural basis for selecting tuber starches in food processing, where PS may be suitable for applications requiring high energy input for gelatinization, while SPS and TS may be preferred when higher gelatinization temperatures are needed to maintain texture during heating.

### 2.5. Textural Properties

The textural properties of starch gels, including hardness, adhesiveness, springiness, cohesiveness, gumminess, chewiness, and resilience, are presented in Table 5. These parameters are critical indicators of gel network strength and structural integrity, and are directly influenced by the molecular architecture of starch, particularly the amylose content, amylopectin chain-length distribution, and granule morphology [29]. PS exhibited significantly higher values for hardness (519.31 g), cohesiveness (0.92), gumminess (477.98 g), chewiness (465.49 g), and resilience (0.83) compared with SPS (hardness 110.29 g, cohesiveness 0.86, gumminess 95.33 g, chewiness 94.96 g, resilience 0.79) and TS (hardness 100.29 g, cohesiveness 0.84, gumminess 84.41 g, chewiness 76.66 g, resilience 0.78). In contrast, SPS showed the highest springiness (1.00), whereas PS and TS gave lower values (0.97 and 0.91, respectively), although the differences were not statistically significant. The PS gel exhibited markedly higher hardness, gumminess, and chewiness than the SPS and TS gels, indicating a firmer and more cohesive gel texture. This behavior is consistent with the RVA results, where PS exhibited the highest peak viscosity and final viscosity, reflecting a paste with strong inter-chain associations upon cooling. Several molecular factors contribute to this superior gel strength of PS. First, PS has the highest proportion of medium and long amylopectin chains (DP 13–24, 25–36 and 37–100), which facilitate the formation of stable intermolecular junctions and reinforce the gel network [20,30]. Second, the relatively low amylose content of PS (21.98%) allows greater swelling of granules during gelatinization, enabling more extensive entanglement of leached amylose and amylopectin chains upon cooling [31]. Our results are broadly consistent with a previous study on yam starch, which observed that higher textural parameters were associated with larger starch granule size and lower amylose content [32].

Additionally, the relatively short amylopectin chains in SPS and TS are less effective in forming stable crystalline junctions, thereby reducing the resistance of the gel network to deformation. Although SPS contains a relatively high proportion of medium-length amylose chains (DP 500–5000), which may favor retrogradation, as reflected by its high setback viscosity. This characteristic did not translate into superior gel hardness in the texture profile analyzer (TPA) measurement. This apparent discrepancy can be explained by the fact that setback viscosity primarily reflects the early stage of amylose retrogradation and short-range ordering [33], whereas TPA hardness depends on the overall network architecture, junction density, and the ability of the final gel to resist deformation. The higher hardness of PS gels may therefore be governed by other factors, such as its longer amylopectin chains and/or phosphate monoesters, which promote granule swelling and the formation of a more continuous and rigid gel network.

The cohesiveness, gumminess, and resilience values followed a trend similar to that of hardness, with PS exhibiting the highest values, indicating that PS gels were not only firmer but also more well-structured and better able to recover from deformation. The resilience values, which reflect the elastic recovery of the gel after compression, were slightly higher for PS (0.83) than for SPS (0.79) and TS (0.78), though the differences were not statistically significant. This suggests that the gel network of PS has a higher degree of elasticity, possibly due to the longer amylopectin chains that act as flexible cross-linkers between crystalline junctions [27].

In summary, the TPA results demonstrate that PS gels possess superior mechanical strength and cohesiveness compared with SPS and TS, which may be attributed to PS’s unique combination of large granule size (Figure S1), high medium-chain amylopectin content, and low amylose content. These findings provide practical guidance for the selection of tuber starches in food formulations: PS is well-suited for applications requiring firm, elastic gels (e.g., confectionery, thick sauces, and processed meat products), while SPS and TS, with their softer gels, are more appropriate for products where a delicate texture is desired (e.g., puddings, custards, or as stabilizers in dairy products).

### 2.6. Freeze–Thaw Stability

The freeze–thaw stability of starch gels was evaluated by measuring the syneresis rate (SR, %), which reflects the extent of water separation upon repeated freezing and thawing. A lower SR indicates better freeze–thaw stability, as the gel network retains more water and resists structural collapse [34]. As shown in Table 5, PS exhibited the highest SR (67.07%), followed by TS (60.63%) and SPS (51.60%). Statistical analysis (Tukey’s HSD test) revealed that PS had significantly higher SR than SPS, while TS showed intermediate values that were not statistically different from either PS or SPS. These results indicate that SPS possesses superior freeze–thaw stability among the three starches, whereas PS is the most susceptible to syneresis. The pronounced differences in syneresis can be rationalized by considering the molecular and structural features of each starch [35]. Freeze–thaw instability is primarily attributed to the retrogradation of amylose and the aggregation of amylopectin chains during frozen storage, which disrupt the gel network and expel water [36]. SPS, which contains a higher proportion of short amylopectin chains (DP 6–12) and medium-length amylose chains (DP 500–5000), appears to form a gel network with a more compact and ordered arrangement that effectively immobilizes water molecules [36], thereby resisting syneresis. This interpretation is consistent with the TPA results, where SPS exhibited moderate hardness but high springiness (1.00), suggesting a resilient network that can accommodate freeze–thaw stress without extensive structural disruption.

In contrast, PS displayed the poorest freeze–thaw stability despite its highest gel hardness and cohesiveness. This apparent contradiction can be explained by the distinct network architecture of PS gels. PS is characterized by a large granule size (Figure S1), a high proportion of medium and long amylopectin chains, and a low amylose content. While these features contribute to strong gel formation through extensive entanglements and intermolecular associations, the resulting gel network is relatively coarse and porous, with large interstitial spaces that allow water to separate more readily upon freezing [37]. Moreover, the low amylose content of PS limits the formation of a stable amylose network that could reinforce the gel against freeze–thaw cycles. During freezing, ice crystal formation exerts mechanical stress on the gel matrix; the coarse network of PS is more prone to rupture, leading to higher syneresis.

From a practical perspective, the freeze–thaw stability of starch is a critical quality attribute for frozen food products, such as frozen desserts, sauces, and ready-to-eat meals. The superior stability of SPS makes it a preferred choice for formulations requiring repeated freeze–thaw cycles, whereas PS, despite its excellent thickening and gelling properties, may require modification (e.g., cross-linking or chemical substitution) to enhance its freeze–thaw resistance for such applications.

### 2.7. In Vitro Digestion Properties

The digestibility profiles of the three tuber starches (PS, SPS, and TS), expressed as the percentages of rapidly digestible starch (RDS), slowly digestible starch (SDS), and resistant starch (RS), are presented in Table 5. SPS exhibited the highest RS content (65.66%), which was significantly higher than that of PS (56.36%). TS showed an intermediate RS level (59.97%) that was not significantly different from either. For SDS, all three starches showed comparable values (~23%), with no significant differences observed among them. Conversely, RDS was highest for PS (20.36%), significantly exceeding that of SPS (10.86%), while TS (17.15%) was intermediate and not significantly different from either. These results indicate that SPS is the most nutritionally favorable among the three starches, owing to its exceptionally high RS content and the lowest RDS, which are associated with a lower glycemic response and improved colonic health benefits.

The above differences in digestibility can be attributed to the distinct molecular and supramolecular structures of each starch [38]. The high RS and low RDS in SPS may be attributed to its intermediate amylose content and its enrichment in short amylopectin chains (DP 6–12), as well as medium-length amylose chains (DP 500–5000). These structural features tend to form retrograded amylose and less stable crystalline structures that are more susceptible to enzymatic attack initially, but ultimately lead to a considerable portion of starch escaping digestion [39]. In contrast, although PS possesses a B-type crystalline pattern (Figure S2), a low amylose content (21.98%), and a high proportion of medium and long amylopectin chains (DP 13–24, 25–36, and 37–100), which theoretically should confer higher resistance, its actual RS content was the lowest and RDS the highest. This discrepancy suggests that the dense packing of double helices and the high ΔH value observed in DSC for PS (13.53 J/g) may not fully translate to enzymatic resistance in this specific in vitro system, or that other structural factors are dominating its rapid digestion kinetics.

The high SDS content in PS and TS (≈23%) suggests that these starches can provide prolonged glucose release, which is desirable for sustained energy supply and glycemic control. However, the relatively high RS content of SPS (65.66%) indicates that a considerable fraction of its starch was not digested under the simplified in vitro enzymatic conditions used in this study. In addition, the physical state of the starch during digestion must be considered, because it strongly determines the apparent RS content [40]. Because native granular starch is generally more resistant to enzymatic hydrolysis than gelatinized starch, the apparent RS, SDS, and RDS values obtained here primarily reflect the susceptibility of the native starch granules to the simplified in vitro enzymatic system, rather than the digestion behavior of cooked or processed starch in a food matrix. Consequently, the relatively high RS content of SPS (65.66%) indicates that a considerable fraction of its native starch granules escaped digestion under these conditions. This finding suggests that SPS may have potential as a source of resistant starch for fiber-enriched products, but it does not by itself demonstrate a low glycemic index in finished foods. Likewise, although the high RS content of SPS warrants further investigation, the present data does not provide evidence for prebiotic functionality or colonic health benefits. The moderate SDS levels in PS and TS suggest a possible contribution to more moderate glucose release in the native starch state, whereas PS, despite its firmer gel texture and higher RDS, may still be suitable for applications where a more rapid glycemic response is acceptable, provided that the effects of cooking and food processing are evaluated. These digestion results are descriptive and should not be interpreted as establishing robust structure–property or structure–nutrition relationships. Future studies using multiple cultivars, suppliers, and processing histories, together with gelatinized/cooked starch systems, food-matrix, and in vivo experiments, are needed to validate these preliminary observations.

## 3. Conclusions

In this study, PS, SPS, and TS were systematically characterized from molecular architecture to processing performance, and their possible associations were further discussed. PS was distinguished by higher proportions of medium- and long-chain amylopectin (DP 13–24, 25–36 and 37–100) and lower amylose content, while SPS and TS were enriched in short amylopectin chains (DP 6–12) and had higher amylose contents (TS showing the highest at 28.11%) in this study. These molecular differences translated into distinct functional behaviors: PS exhibited the highest pasting viscosities, gel hardness, gumminess, and chewiness, but the poorest freeze–thaw stability and lower gelatinization temperatures. PS had a relatively low RS content (56.36%) with no significant differences in SDS (~23%). SPS showed the strongest setback viscosity, the best freeze–thaw resistance, the highest RS (65.66%), and the lowest RDS (10.86%), while its gel hardness (110.29 g) did not differ significantly from that of TS (100.29 g), making TS the numerically softest sample. TS exhibited intermediate texture and digestibility properties, with intermediate RDS (17.15%) and RS (59.97%) values that were not significantly different from either extreme, along with the highest lightness. Overall, the results of this study indicate that the functional properties and digestion behavior of three tuber starches (PS, SPS, and TS) are not determined solely by amylose content, but are also influenced by the fine chain-length distributions of both amylopectin and amylose. However, given the limited number of commercial samples, these findings should be regarded as exploratory, and further studies with multiple cultivars and suppliers are needed to confirm these associations. Additionally, future work with a broader range of samples is necessary to establish intrinsic botanical-source effects. Further, research focused on the effects of compatible preservatives should be performed.

## 4. Materials and Methods

### 4.1. Materials

Potato starch (PS) was sourced from Meelunie B.V. (Amsterdam, The Netherlands), while sweet potato starch (SPS) was obtained from Hubei Xiangjie Shipin Co., Ltd. (Jingmen, China), and tapioca starch (TS) was purchased from Bangkok International Food Co., Ltd. (Koh Samui, Thailand). The enzymes α-amylase (P7545), amyloglucosidase (A7095), and the glucose oxidase–peroxidase (GOPOD) reagent were procured from Sigma-Aldrich Ltd. (St. Louis, MO, USA). A total starch assay kit (K-TSTA) and isoamylase (EC 3.2.1.68) were acquired from Megazyme International Ltd. (Bray, Co., Wicklow, Ireland). Pullulan standards with peak molecular weights between 180 Da and 1.2 × 106 Da, for the SEC calibration curve, were obtained from Polymer Standards Service (PSS, Mainz, Germany). All other chemicals were of analytical grade and used without further purification.

### 4.2. Total Starch and Color Measurement

Total starch content was determined with a total starch assay kit (K-TSTA) following the manufacturer’s protocol and our previous description [41]. Color attributes (L*, a*, b*) were determined using a colorimeter (Caipu Technology Co., Ltd., Hangzhou, China) calibrated with a standard whiteboard (97.6 of L*, 0.2 of a*, 1.3 of b*), following the manufacturer’s instructions.

### 4.3. Chain-Length Distribution (CLD) Measurement

Starch samples (4–6 mg) were dissolved in 1.5 mL DMSO/LiBr (0.5% *w*/*w*) at 80 °C for 24 h with stirring, followed by centrifugation at 4000× *g* for 10 min. The supernatant was precipitated twice with 10 mL absolute ethanol to remove lipids and other soluble impurities. The precipitated starch was placed in 0.9 mL of warm deionized water, heated in a boiling water bath for 2 h to gelatinize the starch, and then cooled to room temperature. For CLD analysis, the gelatinized starch was debranched with isoamylase at a final concentration of 2.5 Units/mL in 50 mM sodium acetate buffer (pH 4.5) at 37 °C for 24 h with gentle shaking. The reaction was terminated by neutralization to pH ~7 using 0.1 M NaOH, followed by freeze-drying. The freeze-dried debranched samples were then redissolved in DMSO/LiBr (4 mg/mL) and transferred to fluorophore-assisted carbohydrate electrophoresis (FACE) and size-exclusion chromatography (SEC), following a protocol established in our laboratory [15,16].

FACE analysis: the debranched starch samples were labelled with 8-aminopyrene-1,3,6-trisulfonic acid (APTS) prior to electrophoretic separation, according to standard procedures. The resulting chain profiles were acquired on a PA-800 Plus FACE system (Beckman-Coulter, Brea, CA, USA), equipped with an argon-ion excitation laser and a solid-state laser-induced fluorescence detector. Separations were performed in an N-CHO-coated capillary (total length 40 cm, inner diameter 50 µm) using the commercial running buffer supplied with the Carbohydrate Labeling and Analysis Kit. Sample injection was carried out by pressure-assisted application at 0.5 psi for 3 s, and electrophoresis was run at a constant voltage of 30 kV and a controlled temperature of 25 °C.

SEC analysis: the debranched samples were redissolved in 0.5% (*w*/*v*) LiBr/DMSO at a concentration of 4 mg/mL and then fractionated on a column set comprising a GRAM pre-column, a GRAM 100 column, and a GRAM 1000 column (PSS, Mainz, Germany). The SEC runs were carried out at 80 °C with a mobile-phase flow rate of 0.6 mL/min. SEC characterization was performed on an LC-20AD Shimadzu SEC system (Agilent, Santa Clara, CA, USA) with a refractive index detector (RID-20A, Shimadzu Corporation, Kyoto, Japan). The DP was determined from *R*_h_ by the Mark–Houwink equation with a series of pullulan standards for calibration, in which the parameters *K* and *a* of linear glucose polymers in DMSO at 80 °C (the SEC eluent and elution temperature) were taken as 5 × 10^−4^ dL g^−1^ and 0.743, respectively. Amylose content was obtained by finding the ratio of the area under the amylose region divided by the total area in the debranched SEC data, which is at the degree of polymerization (~100) where a clear separation of amylose and amylopectin chains is seen.

### 4.4. Rapid Viscosity Analyzer (RVA) Measurement

Pasting profiles of starch samples were recorded using a Rapid Visco Analyzer (RVA-TecMaster, Perten Instruments Australia Pty Ltd., Macquarie Park, NSW, Australia) according to a previously described procedure [22]. Briefly, 1.5 g of starch (dry basis) was accurately weighed and thoroughly mixed with 25 g of deionized water directly in the RVA test canister. The slurry was equilibrated at 50 °C for 1 min, heated to 95 °C at a rate of 12 °C/min, held at that temperature for 2.5 min, then cooled to 50 °C at the same rate and finally maintained at 50 °C for an additional 2 min. The agitation speed was set at 960 rpm during the first 10 s to ensure complete dispersion, and subsequently reduced to 160 rpm for the remainder of the assay.

### 4.5. Differential Scanning Calorimetry (DSC) Measurement

Thermal profiles of starch samples were evaluated using a differential scanning calorimeter (DSC Q2000, TA Instruments Inc., New Castle, DE, USA) following a previously described procedure [42]. In brief, each dried sample (3 mg) was weighed into an aluminum pan and hydrated with 9 μL of distilled water. The pan was hermetically sealed and stored at 4 °C for 24 h to ensure complete moisture equilibration, then brought to room temperature prior to analysis. The heating scan was conducted from 30 °C to 100 °C at a constant rate of 10 °C/min, with an identical empty pan serving as the reference.

### 4.6. Texture Profile Analyzer (TPA) Measurement

After the RVA test described in Section 4.4, the resulting starch pastes were individually transferred into 9-mL cubic molds and stored at −4 °C for 24 h for cold storage and retrogradation. The resulting solid starch gels were then used as the samples for TPA. Textural measurements were performed on a TA.XT Plus Texture Analyzer (Stable Micro Systems, Godalming, UK) fitted with a 36 mm cylindrical probe. All tests were performed at a compression rate of 50%, a compression cycle of 2, and a whole test speed of 1 mm/s in accordance with previously reported methods [43].

### 4.7. Freeze–Thaw Measurement

The freeze–thaw stability of each starch sample was assessed using a modified method from Yang et al. (2025) [35]. A 5% (*w*/*w*) aqueous starch suspension was first prepared and fully gelatinized by continuous heating in a boiling water bath for 30 min. After cooling to ambient temperature, 30 g of the resulting paste was weighed into a 50 mL centrifuge tube. The capped tubes were kept at −18 °C for 18 h and then allowed to thaw spontaneously at 25 °C for 6 h. This freezing–thawing cycle was repeated for three times. The mass of water release (g) after three cycles (based on the total starch paste mass; 30 g) was expressed as the syneresis rate (SR; g/g).

### 4.8. In Vitro Digestion Measurement

The digestibility of starch samples was evaluated using an in vitro enzymatic assay [44]. Briefly, 100 mg of dry starch was incubated at 37 °C in 10 mL of 0.2 M sodium acetate buffer (pH 5.2) containing 290 units/mL porcine pancreatic α-amylase and 15 units/mL amyloglucosidase. At time points of 0, 20 and 120 min, aliquots of 0.5 mL were withdrawn from the reaction mixture and promptly mixed with 2 mL of absolute ethanol to stop the enzymatic reaction. The liberated glucose was quantified using a GOPOD assay kit, and the percentage of starch hydrolysis was derived by applying a conversion factor of 0.9 to the glucose values. Percentages of rapidly digestible starch (RDS, % digestible starch at 20 min − % digestible starch at 0 min), slowly digestible starch (SDS, % digestible starch at 120 min − % digestible starch at 20 min), and resistant starch (RS, 100% − % digestible starch at 120 min) were calculated and normalized to the total starch content [45].

### 4.9. Statistical Analysis

All experiments were performed in triplicate or more, and the results were expressed as mean ± standard deviation (SD). Statistical evaluation was carried out by one-way analysis of variance (ANOVA) with Tukey’s post hoc test for multiple comparisons, using SPSS Statistics 25 (IBM Corp., Armonk, NY, USA). A probability value of *p* < 0.05 was considered statistically significant.

## Figures and Tables

**Table 1:** Total starch content and chroma values of three tuber starches (PS, SPS, and TS).

Parameter	Potato Starch (PS)	Sweet Potato Starch (SPS)	Tapioca Starch (TS)
Total starch content (%)	94.03 ± 3.09 ^a^	92.14 ± 1.12 ^a^	92.56 ± 0.87 ^a^
L*	96.37 ± 0.15 ^b^	96.57 ± 0.15 ^b^	98.30 ± 0.10 ^a^
a*	0.70 ± 0.00 ^b^	1.00 ± 0.00 ^a^	0.72 ± 0.13 ^b^
b*	1.40 ± 0.10 ^b^	3.37 ± 0.06 ^a^	1.24 ± 0.09 ^b^

Different superscript letters were used to represent statistical significance among different samples (*p* < 0.05).

**Table 2:** Chain-length distributions (CLDs) of amylopectin and amylose in three tuber starches (PS, SPS, and TS).

Chain Length Ratio (%)	Potato Starch (PS)	Sweet Potato Starch (SPS)	Tapioca Starch (TS)
Amylopectin (AP)			
DP 6–12	32.59 ± 0.94 ^b^	41.02 ± 0.01 ^a^	41.72 ± 0.02 ^a^
DP 13–24	40.80 ± 1.87 ^a^	36.92 ± 0.01 ^b^	37.55 ± 0.08 ^ab^
DP 25–36	10.78 ± 0.39 ^a^	9.71 ± 0.02 ^b^	9.40 ± 0.01 ^b^
DP 37–100	15.84 ± 0.54 ^a^	12.35 ± 0.01 ^b^	11.33 ± 0.04 ^c^
Amylose (AM)			
DP 100–500	21.14 ± 0.45 ^b^	9.90 ± 0.27 ^c^	23.44 ± 0.57 ^a^
DP 500–5000	69.48 ± 0.06 ^b^	81.34 ± 1.00 ^a^	70.47 ± 1.11 ^b^
DP 5000–20,000	9.56 ± 0.31 ^a^	8.97 ± 0.68 ^a^	6.44 ± 0.78 ^b^
AM content (%)	21.98 ± 0.39 ^b^	22.05 ± 0.26 ^b^	28.11 ± 0.03 ^a^

Different superscript letters were used to represent statistical significance among different samples (*p* < 0.05).

**Table 3:** RVA characteristic values of three tuber starches (PS, SPS, and TS).

Parameter	Potato Starch (PS)	Sweet Potato (SPS)	Tapioca Starch (TS)
PV (cP)	6876.00 ± 155.23 ^a^	3133.50 ± 47.37 ^b^	2870.50 ± 26.16 ^b^
TV (cP)	3174.67 ± 85.33 ^a^	1813.50 ± 41.72 ^b^	1376.00 ± 16.97 ^c^
BD (cP)	3701.33 ± 93.18 ^a^	1320.00 ± 5.65 ^c^	1494.50 ± 9.19 ^b^
FV (cP)	3505.00 ± 56.63 ^a^	2526.00 ± 14.14 ^b^	1879.50 ± 26.16 ^c^
SB (cP)	330.33 ± 38.76 ^c^	712.50 ± 55.86 ^a^	503.50 ± 43.13 ^b^
PT1 (min)	4.42 ± 0.14 ^ab^	4.37 ± 0.09 ^bc^	4.54 ± 0.09 ^a^
PT2 (℃)	78.57 ± 2.27 ^b^	79.13 ± 1.17 ^b^	81.60 ± 1.20 ^a^

Note: PV, peak viscosity; TV, trough viscosity; BD, breakdown viscosity; FV: final viscosity; SB: setback viscosity, PT1: peak time; PT2: pasting temperature. Different superscript letters were used to represent statistical significance among different samples (*p* < 0.05).

**Table 4:** DSC characteristic values of three tuber starches (PS, SPS, and TS).

Parameter	Potato Starch (PS)	Sweet Potato (SPS)	Tapioca Starch (TS)
*T*_o_ (℃)	64.27 ± 0.21 ^a^	62.97 ± 5.25 ^a^	66.10 ± 0.20 ^a^
*T*_p_ (℃)	67.97 ± 0.29 ^b^	70.93 ± 0.21 ^a^	70.53 ± 0.12 ^a^
*T*_c_ (℃)	73.00 ± 0.46 ^b^	77.23 ± 0.47 ^a^	77.13 ± 0.12 ^a^
∆*H* (J/g)	13.53 ± 0.93 ^a^	10.69 ± 0.06 ^b^	11.24 ± 0.63 ^b^
∆*T* (℃)	8.73 ± 0.31 ^b^	14.27 ± 5.58 ^a^	11.03 ± 0.11 ^a^

Note: *T*_o_, onset temperature; *T*_p_, peak temperature; *T*_c_, conclusion temperature; ∆*H*, enthalpy change; ∆*T*, temperature range = *T*_c_ − *T*_o_. Different superscript letters were used to represent statistical significance among different samples (*p* < 0.05).

**Table 5:** Textural, freeze–thaw, and in vitro digestion characteristic values of three tuber starches (PS, SPS, and TS).

Parameter	Potato Starch (PS)	Sweet Potato (SPS)	Tapioca Starch (TS)
Texture			
Hardness (g)	519.31 ± 46.69 ^a^	110.29 ± 3.07 ^b^	100.29 ± 3.01 ^b^
Adhesiveness (g·s)	−21.61 ± 11.71 ^a^	−4.23 ± 4.64 ^a^	−11.85 ± 1.69 ^a^
Springiness	0.97 ± 0.01 ^a^	1.00 ± 0.00 ^a^	0.91 ± 0.07 ^a^
Cohesiveness	0.92 ± 0.02 ^a^	0.86 ± 0.02 ^b^	0.84 ± 0.03 ^b^
Gumminess (g)	477.98 ± 45.99 ^a^	95.33 ± 2.18 ^b^	84.41 ± 5.81 ^b^
Chewiness (g)	465.49 ± 46.88 ^a^	94.96 ± 1.87 ^b^	76.66 ± 10.38 ^b^
Resilience	0.83 ± 0.03 ^a^	0.79 ± 0.03 ^a^	0.78 ± 0.06 ^a^
Freeze–thaw			
SR (%)	67.07 ± 4.03 ^a^	51.60 ± 7.05 ^b^	60.63 ± 1.25 ^ab^
Digestion			
RDS (%)	20.36 ± 2.27 ^a^	10.86 ± 0.56 ^b^	17.15 ± 4.08 ^ab^
SDS (%)	23.28 ± 1.51 ^a^	23.48 ± 0.60 ^a^	22.88 ± 3.55 ^a^
RS (%)	56.36 ± 3.64 ^b^	65.66 ± 0.52 ^a^	59.97 ± 7.19 ^ab^

Note: SR, syneresis rate; RDS, rapidly digestible starch; SDS, slowly digestible starch; RS, resistant starch. Different superscript letters were used to represent statistical significance among different samples (*p* < 0.05).

## Data Availability

The datasets generated for this study are available on request to the corresponding author.

## Supplementary Materials

The following supporting information can be downloaded at: https://www.mdpi.com/article/10.3390/gels12100939/s1, Table S1: Moisture, protein, lipid, ash content and lot information of three tuber starches (PS, SPS, and TS); Figure S1: Microscopic morphology and particle size distribution of three tuber starches (PS, SPS, and TS); Figure S2: X-ray diffraction pattern of three tuber starches (PS, SPS, and TS).
