# Supplementary material for: A Systematic Comparative Study of Potato, Sweet Potato, and Tapioca Starches: From Molecular Architecture to Processing Performance
Source: Gels. 2026 Oct 20;12(10):939. doi: 10.3390/gels12100939 (PMC13704648; doi:10.3390/gels12100939)
Supplement: Supplementary file 1 [file gels-12-00939-s001.zip › gels-4576064-supplementary.pdf]

Supplemental data

A systematic comparative study of potato, sweet potato, and tapioca starches: From molecular architecture to processing performance

Table S1. Moisture, protein, lipid, ash content and lot information of three tuber starches (PS, SPS, and TS)

Parameter	Potato starch (PS)	Sweet potato starch (SPS)	Tapioca starch (TS)
Moisture content (%) [A]	18.32 ± 0.05^{a}	12.38 ± 0.08^{b}	12.01 ± 0.09^{c}
Protein content (%) [B]	0	0	0
Lipid content (%) [B]	0	0.7	0
Ash content (%) [B]	0.012	0.022	0
Lot information [B]	XD212B7K2EI03	6974312280112	8850341300206

Different superscript letters were used to represent statistical significance among different samples ($p < 0.05$).

[A] The measurement for moisture content is determined through the Mettler HE53 moisture analyzer (Mettler-Toledo, LLC, Columbus, OH, USA).

[B] This data is collected from the sample package.

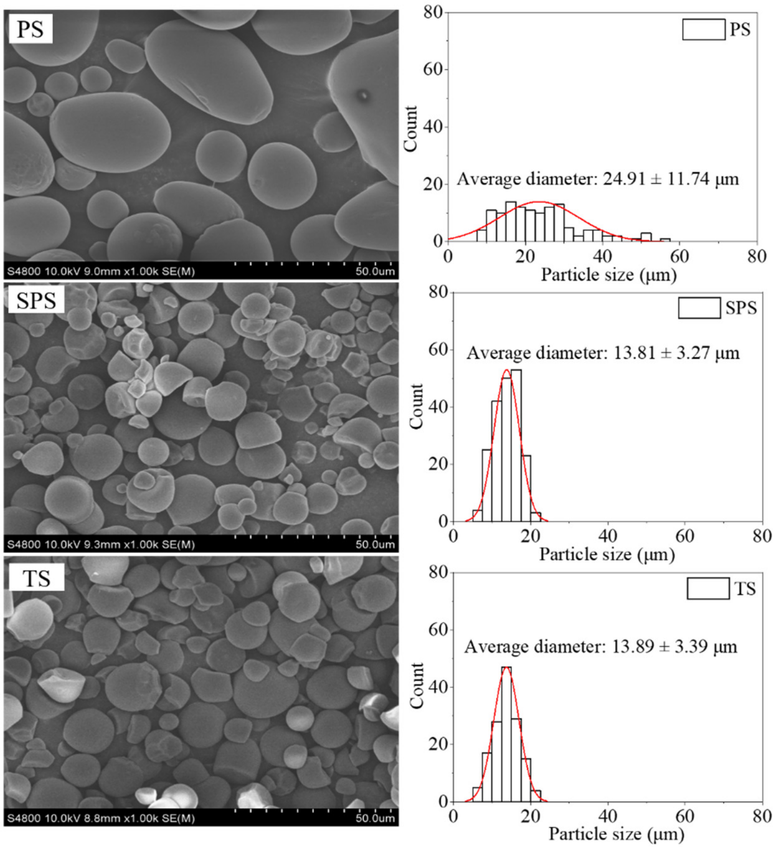

Figure S1. Microscopic morphology and particle size distribution of three tuber starches (PS, SPS, and TS)

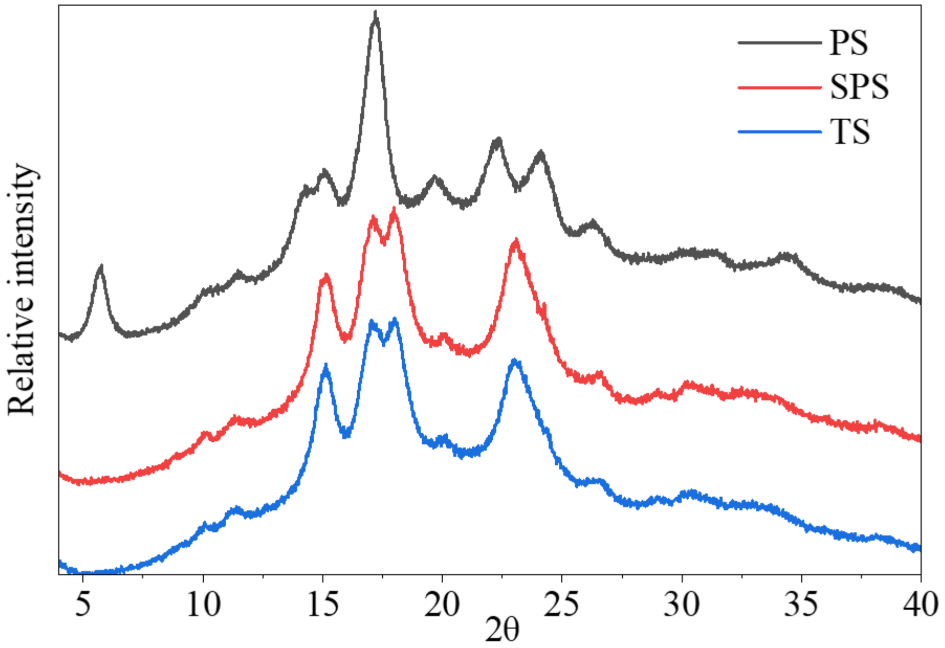

Figure S2. X-ray diffraction pattern of three tuber starches (PS, SPS, and TS)
